# Very late-onset complete atrioventricular block following deployment of Amplatzer membranous ventricular septal defect occluder

**DOI:** 10.1097/MD.0000000000018412

**Published:** 2019-12-20

**Authors:** Shuran Shao, Chunyan Luo, Kaiyu Zhou, Yimin Hua, Chuan Wang

**Affiliations:** a Department of Pediatric Cardiology, West China Second University Hospital, Sichuan University,; b The Cardiac development and early intervention unit, West China Institute of Women and Children's Health, West China Second University Hospital, Sichuan University,; c Department of Radiology, West China Hospital, Sichuan University, Chengdu, Sichuan, China; d Key Laboratory of Birth Defects and Related Diseases of Women and Children (Sichuan University), Ministry of Education; e Key Laboratory of Development and Diseases of Women and Children of Sichuan Province, West China Second University Hospital, Sichuan University, Chengdu, Sichuan, China.

**Keywords:** CAVB, children, occluder, PmVSD

## Abstract

**Rationale:**

Although implantation of Amplatzer membranous ventricular septal defect occluder (AVSDO) is an alternation to surgical treatment, the interventional therapy is disapproved by FDA due to high incidence of complete atrioventricular block (cAVB) post closure during early and middle term follow-up. However, long-term outcomes of the accumulating numbers of patients who had received AVSDO in the past decades, still remain an issue of concern and late occurrence of potentially catastrophic heart block long after hospital discharge is especially worrying, but rarely documented. We firstly reported a pediatric case with very late-onset cAVB occurring over ten years following transcatheter closure of PmVSD using AVSDO.

**Patient concerns:**

A 5-year old female received transcatheter closure of PmVSD sized 10-mm on left ventricular angiography with a 14-mm AVSDO owning to a history of recurrent lower respiratory tract infections. Post-procedure echocardiography documented no arrhythmias, residual shunt and aortic regurgitation. All electrocardiogram (ECG) recordings were completely normal and transthoracic echocardiography (TTE) examination showed the device was in the proper position and there was neither residual shunt nor valves regurgitation. Ten years after operation, the patient was re-admitted into our hospital due to recurrent syncope.

**Diagnoses:**

A 12-lead ECG showed cAVB with a minimal heart rate of 42 bpm. Device flattening was revealed on 2-dimensional TTE and the occluder appeared to return to its original size and shape. Computed tomography and magnetic resonance imaging of brain did not reveal any intracranial hemorrhages, ischemic changes, or space-occupying lesions. Electroencephalogram detected no epileptiform discharge. Other possible etiologies resulting in cAVB such as myocarditis, hypothyroidism and connective tissue diseases were excluded. Therefore, it was ultimately considered the cAVB was mostly likely to be associated with device closure of PmVSD using AVSDO.

**Interventions:**

The child was empirically treated with prednisone (1–2 mg/Kg daily).

**Outcomes:**

Unfortunately, no improvement was observed. A permanent pacemaker was implanted. The following course was uneventful.

**Lessons:**

For patients following transcatheter closure of PmVSD using AVSDO, the risk period for developing heart block after device closure appears to be much longer than we speculated. Long-term, perhaps and life-long followed up needs to be considered for this group of patients.

## 1 Introduction

Transcatheter closure of perimembranous ventricular septal defect (PmVSD) with Amplatzer membranous VSD occluder (AVSDO) is an alternation to surgical treatment in selected patients with high closure rate and low mortality in the early decade of 21st century.^[1–4]^ However, a high incidence of complete atrioventricular block (cAVB) post closure, ranging from 8.7% to 20% during early and middle term follow-up,^[3,5]^ was documented, leading to the disapproval of this interventional therapy by FDA. Despite implantation of AVSDO was terminated by most centers thereafter, long-term outcomes of the accumulating numbers of patients who had received AVSDO in the past decades, still remain an issue of concern. The late occurrence of potentially catastrophic heart block long after hospital discharge is especially worrying, but rarely documented.^[3,5–8]^ Herein, we first reported a case with very late-onset cAVB occurring over 10 years following transcatheter closure of PmVSD using AVSDO, highlighting the importance of long-term follow-up for these patients and providing some explanations for the exact mechanism of late cAVB.

## 2 Ethics statements

Informed written consent was obtained from the parents after the nature of this study had been fully explained to them. The parents of patient have provided informed consent for publication of the case.

## 3 Case report

A 5-year old female weighing 17 Kg, with a PmVSD and a history of recurrent lower respiratory tract infections, was referred to our hospital for transcatheter closure of the defect. Transthoracic echocardiography (TTE) revealed a 10-mm sized defect with a left to right shunt, moderate pulmonary hypertension (estimated systolic pulmonary arterial pressure: 50 mmHg) and left ventricle enlargement (end-diastolic dimension: 38 mm). Informed consent to the procedure was obtained from the child's parents. The procedure was undertaken under general anesthesia and performed in a standard way detailed in our previous study.^[9]^ The defect measured 9.5 mm on left ventricular angiography and a 14-mm AVSDO (AGA Medical, Golden Valley, Minn) was chosen. No arrhythmias, residual shunt and aortic regurgitation was documented following occluder deployment and the device was released. Oral administration of aspirin (75 mg daily) was initiated and the child was subjected to 72 hours of dynamic ECG monitoring, as well as a 12-lead ECG and echocardiography at 1, 3, 7 days post procedure, during which time the patient was uneventful and discharged 1 week later.

**Figure 1:**
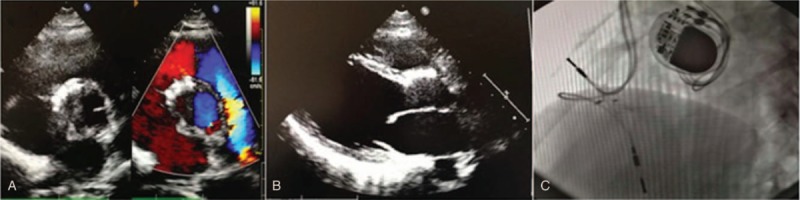
Findings on TTE and fluoroscopic evaluation over ten years post device closure. (A) parasternal short-axis view on TTE, (B) parasternal long-axis view on TTE, (C) fluoroscopic appearance of the device. TTE = Transthoracic echocardiography.

After discharge, the patient was followed up clinically as well as with 24-hour dynamic ECG, and echocardiography at 1, 3, 6, and 12 months during the first year and annually thereafter. All the ECG recordings were completely normal and TTE examination showed the device was in the proper position and there was neither residual shunt nor valves regurgitation. Ten years after operation, the patient was re-admitted into our hospital due to recurrent syncope. Any history of drug or toxins exposure was completely denied. On arrival, he was clear, afebrile, no tachypnea and no hypotension. The vital signs were stable, and no positive findings were identified by physical examination expect for a slow heart rate (40–50 bpm). Blood routine test, blood electrolytes, acute phase protein, blood glucose, blood gas analysis, liver function and creatinine level were unremarkable. Computed tomography and magnetic resonance imaging of brain did not reveal any intracranial hemorrhages, ischemic changes, or space-occupying lesions. Electroencephalogram detected no epileptiform discharge. Device flattening was revealed on 2-dimensional TTE and the occluder appeared to return to its original size and shape (Fig. 1A-B), no other abnormal findings were noted. A 12-lead ECG showed cAVB with a minimal heart rate of 42 bpm. Thereafter, several other examinations including myocardial troponin, thyroid function, autoantibody and antineutrophil cytoplasmic antibody (ANCA) were undergone to further explore the underlying etiology resulting in cAVB, but all of them were normal. Based on a history of device closure of PmVSD with AVSDO, device flattening on TTE and exclusion of other possible etiologies resulting in cAVB such as myocarditis, hypothyroidism and connective tissue diseases, it was ultimately considered the cAVB was mostly likely to be associated with device closure of PmVSD using AVSDO. The child was empirically treated with prednisone (1–2 mg/Kg daily), but no improvement was observed. Lastly, a permanent pacemaker was implanted and device flattening was also revealed on fluoroscopic evaluation (Fig. 1C). The following course was uneventful.

## 4 Discussion

A relatively higher incidence of cAVB after transcatheter closure of PmVSD using AVSDO has been documented in previous studies.^[3–5]^ Butera and colleagues^[3]^ reported the outcomes of 104 patients following device closure of PmVSD using AVSDO with a median follow-up of 38.5 months. The incidence of cAVB was 8.7%, and a permanent pacemaker was required in 6 (5.7%) patients, 2 in the early phase and 4 during late follow-up. Thereafter, Predescu et al^[5]^ reported 4 out of 18 patients (22%) suffered from cAVB at 17 days, 4.2 months, 8.8 months, and 37.5 months following implantation, respectively. Another study^[4]^ from China enrolling 294 patients revealed the incidence of post-procedure cAVB was 5.8% (17/294), with 15 early cAVB, 3 late cAVB, and 1 patient with early and late cAVB. On the basis of these data, implantation of AVSDO was terminated and abandoned worldwide since AV block and permanent pacemaker was only observed in 1.1% of 4432 patients with PmVSD surgical repair.^[10]^

However, all of aforementioned studies only focused on the early and midterm follow-up results of these patients and it seemed that the long-term outcomes raised less attention from clinicians. The published reports on this complication described the latest onset of cAVB presenting at 5 years post implantation.^[3–8]^ To our knowledge, we first reported the latest onset of cAVB occurring over ten years following transcatheter closure of PmVSD using AVSDO. This case report provided important evidences that the at-risk period for developing heart block after device closure appears to be much longer than we speculated, which is similar to that following surgical repair.^[10]^ Most importantly, this finding was of clinically amount significance for underscoring the importance and necessity of long-term, perhaps life-long follow-up for these patients, since thousands of patients receiving transcatheter closure of PmVSD with this kind of device had been documented in the literature.^[1]^ The fact should be considered when counseling the patients and their parents, avoiding irregular or lost follow-up.

The exact mechanism of late cAVB remains speculative. Yalonetsky et al^[8]^ previously reported 1 case with late-onset cAVB occurring 3 years post procedure and suggested a progressive ongoing inflammatory process in the conduction tissue may be one cause of late cAVB owing to its late response to corticosteroid therapy. However, the present case did not support the theory. Another proposed explanation is that fibrosis around the area of the device might extend into the AV node and impair conduction. Additionally, progressive device flattening of an originally oversized occluder may account for the late cAVB. Yang et al^[11]^ reported that 1 patient who developed transient cAVB 7 days after device closure had recurrent cAVB 42 months after the procedure. During the follow-up, the shape of the device returned gradually to its original size and shape, which may compress and cause persistent damage for conduction tissue, leading to the occurrence of late cAVB. For the present case, the VSD size was 9.5 mm on left ventricular angiography and an oversized AVSDO (14-mm) was chosen, which may lead to the progressive device flattening and account for the very late-onset cAVB.

## 5 Conclusion

In conclusion, cAVB could occur over 10 years following transcatheter closure of PmVSD using AVSDO. The at-risk period for developing heart block after device closure appears to be much longer than we speculated. Long-term, perhaps and life-long followed up needs to be considered for this group of patients. The exact mechanism of late cAVB remains speculative. Progressive device flattening may partially contribute to the occurrence of very late-onset cAVB.

## Author contributions

**Resources:** Chunyan Luo, Kaiyu Zhou, Yimin Hua, Chuan Wang.

**Supervision:** Kaiyu Zhou, Yimin Hua, Chuan Wang.

**Writing – original draft:** Shuran Shao.

**Writing – review & editing:** Shuran Shao, Chunyan Luo, Chuan Wang.
